# The postictal systems window hypothesis of electroconvulsive therapy in major depression

**DOI:** 10.3389/fpsyt.2026.1889207

**Published:** 2026-08-12

**Authors:** Bernard Rybczynski, Maciej Maslyk, Bartosz Wojciech Maj, Michal Pruc, Iwona Niewiadomska, Lukasz Szarpak

**Affiliations:** 1 6th Department of Psychiatry, Mazovian Specialist Health Centre in Pruszkow, Pruszkow, Poland; 2 Institute of Biological Sciences, The John Paul II Catholic University of Lublin, Lublin, Poland; 3 Department of Internal Medicine, Health Center of Tomaszow Mazowiecki, Tomaszow Mazowiecki, Poland; 4 Institute of Medical Science, The John Paul II Catholic University of Lublin, Lublin, Poland; 5 Institute of Psychology, The John Paul II Catholic University of Lublin, Lublin, Poland

**Keywords:** biomarkers, cognition, electroconvulsive therapy, HPA axis, inflammation, major depressive disorder, neurovascular coupling, postictal suppression

## Abstract

Electroconvulsive therapy (ECT) remains the most effective acute treatment for severe major depressive disorder, particularly in melancholic, psychotic, suicidal, catatonic or retarded, and treatment-resistant presentations. Existing mechanistic accounts are biologically valid but are often presented as parallel lists: monoamine release, excitatory-inhibitory rebalancing, neuroendocrine activation, immune modulation, neurotrophic plasticity, hippocampal change, and network rewiring. We propose the postictal systems window hypothesis. The organizing principle is seizure-induced postictal recoupling: the therapeutic seizure interrupts a rigid depressive state, whereas the subsequent recovery phase couples electrophysiological, neurovascular-metabolic, clinical, endocrine, and immune-metabolic dynamics. The central claim is not that ECT affects every biological domain. The claim is that an adequate seizure triggers a timed postictal state in which a smaller set of core events - postictal network interruption and recovery, a structured hemodynamic-metabolic transition, and early clinical reorientation - becomes coupled to endocrine and immune-metabolic signaling and later consolidation. This hierarchy is critical. Neurovascular coupling and hemodynamic waves are treated as core components of the postictal transition; blood-brain barrier and neurovascular-unit permeability are treated as plausible but second-order interfaces, not established human mediators. Glial, trophic, structural, and epigenetic changes are placed mainly downstream as translation and consolidation mechanisms. Recent optical neuroimaging evidence that ECT seizures are followed by hyperemic waves consistent with cortical spreading depolarization supports the principle that the postictal period contains organized biological events rather than simple seizure termination. The model is falsifiable: early postictal curves after the first one to three treatments should predict remission and cognitive burden better as a prespecified composite than as isolated markers such as seizure duration, postictal suppression, cortisol, C-reactive protein, or brain-derived neurotrophic factor. Cognitive adverse effects should relate to excessive duration, delayed recovery, or medial temporal exposure of the window rather than to antidepressant efficacy itself.

## 1 Introduction

### 1.1 The unresolved mechanism of one of psychiatry’s strongest treatments

Electroconvulsive therapy (ECT) occupies an unusual position in psychiatry. It is one of the most effective acute treatments for severe major depressive disorder, yet the field still lacks a mechanistic account proportionate to its clinical effect (1–4). Contemporary guidelines describe ECT as a controlled medical procedure requiring attention to indication, consent, anesthesia, stimulus dosing, electrode placement, seizure adequacy, cognitive monitoring, and continuation care (1, 3). Meta-analytic evidence supports efficacy in depressive disorders, particularly when severity and risk make delay hazardous (1, 5). ECT is therefore not a marginal intervention. It is one of the clearest demonstrations that a malignant depressive state can be altered by a brief, repeated biological intervention (1, 3).

The persistence of mechanistic uncertainty matters clinically. It shapes how psychiatrists discuss ECT with patients and families, how cognitive adverse effects are explained, how parameters are chosen, and how future neuromodulatory treatments are designed (1, 4). Recent mechanistic reviews rightly emphasize that ECT cannot be reduced to either electricity or seizure alone (4–6). Yet the explanatory literature still too often reads as an inventory of affected systems: monoamines, glutamate and gamma-aminobutyric acid (GABA), hypothalamic-pituitary-adrenal (HPA) output, inflammation, neurotrophic factors, hippocampal volume, functional connectivity, glial biology, and gene regulation (1, 6–8). The list is not wrong. Its weakness is that it is insufficiently ordered in time.

The clinical syndrome also argues against a single-pathway explanation. Severe depression is a disorder of mood, cognition, psychomotor function, sleep, appetite, autonomic regulation, endocrine rhythm, threat salience, and self-evaluation (9, 10). In melancholic and psychotic depression these abnormalities are not peripheral correlates; they are the syndrome (10–12). A plausible mechanism of ECT must explain how a time-limited intervention can shift a state stabilized across brain networks and peripheral physiology (6, 13).

### 1.2 Why timing and hierarchy are needed

ECT is not a continuous pharmacological exposure but a series of discrete biological challenges, each with an ictal phase, an immediate postictal phase, a recovery phase, and a longer intersession period during which symptoms, cognition, endocrine output, inflammation, sleep, and behavior may change (1, 7, 14, 15). A convincing model must therefore specify what is triggered by the seizure, what belongs to the postictal state, what consolidates across the course, and what may produce toxicity (14–16).

We propose the postictal systems window hypothesis. The organizing biological principle is seizure-induced postictal recoupling. An adequate therapeutic seizure imposes a brief, high-amplitude perturbation on a depressive state that is otherwise stabilized across neural networks, autonomic-endocrine tone, neurovascular-metabolic support, immune-metabolic signaling, sleep, cognition, and behavior. The clinically relevant event is not the seizure discharge alone, but the organized recovery that follows it: inhibitory rebound and EEG suppression/recovery, restoration of orientation, neurovascular-metabolic matching, autonomic-endocrine activation and termination, and immune-metabolic signaling are proposed to become temporally coupled during the minutes-to-hours after treatment. This coupled recovery state is the postictal systems window. It is the candidate bridge between the immediate seizure and later glial, synaptic, structural, epigenetic, and clinical consolidation (1, 7, 14–20).

The model is hierarchical. The seizure is the necessary trigger; the early postictal transition is the proposed core event; HPA-axis and immune-metabolic responses are coupled modulators; and glial, trophic, structural, and epigenetic processes are positioned mainly downstream as translation and consolidation mechanisms (1, 7, 14, 15, 17–20). BBB/NVU dynamics are retained as exploratory interfaces rather than established human mediators (21, 22). The model therefore asks whether ECT works not by adding a single antidepressant signal, but by repeatedly forcing a maladaptive depressive allostatic state through a constrained recovery process in which reorganization becomes more likely.

The added value of the hypothesis is not that ECT affects many biological systems (4, 6). Its added value is the claim that response and cognitive burden depend on the quality of a timed postictal sequence defined by four dimensions: amplitude, coordination, anatomical distribution, and duration (14, 23). Here, amplitude refers to the magnitude of the acute postictal perturbation; coordination refers to temporal coupling among EEG recovery, reorientation, hemodynamic-metabolic transition, and endocrine or immune-metabolic curves; anatomical distribution refers to which networks and memory-relevant structures are exposed; and duration refers to how long the postictal state persists before recovery or transition to a new equilibrium. These dimensions are not separate mechanisms, but clinically measurable features of the same postictal sequence.

Two seizures of similar duration may therefore produce different postictal suppression, vascular response, endocrine activation, immune-metabolic signaling, reorientation, and outcome (23, 24). Conversely, a technically adequate seizure may fail if the ensuing window is too small, uncoupled, anatomically mistargeted, or prematurely terminated (3, 17). A larger perturbation is not automatically better; the therapeutic target is an optimal window, strong enough to disrupt depressive rigidity, coordinated enough to permit reorganization, and contained enough to limit cognitive toxicity (1, 5, 15, 17).

### 1.3 How the postictal systems window differs from existing network, neuroplasticity, and neuroendocrine models

Established network models of ECT have been essential in moving the field beyond single-transmitter explanations. They describe changes in default-mode, salience, cognitive-control, frontal-limbic, and psychomotor networks, and they plausibly account for the loosening of rigid self-referential cognition, psychotic certainty, suicidal fixation, and psychomotor inhibition. Their limitation is not that they are incorrect, but that they are often spatial rather than temporal. They describe what circuits are reorganized, but they do not fully specify why the minutes-to-hours postictal period after each treatment should carry prognostic information, why recovery of orientation matters clinically, or why endocrine, immune-metabolic, and hemodynamic curves are recruited in the same treatment session.

Neuroplasticity models are also biologically persuasive. ECT-associated changes in BDNF, VEGF, hippocampal and amygdala volume, synaptic remodeling, methylation signatures, and microRNAs provide a plausible route from repeated treatments to durable clinical change. However, these mechanisms are better suited to explaining consolidation across a course than the earliest clinical transitions after one or several treatments. They do not, by themselves, fully explain why suicidal intensity, psychotic preoccupation, psychomotor retardation, or severe depressive rigidity may begin to change before slower structural plasticity can plausibly account for the whole clinical effect. In the present model, neuroplasticity is therefore retained, but positioned downstream as a consolidation mechanism rather than as the primary early mediator.

Similarly, neuroendocrine and inflammatory models identify important systemic components of ECT response, especially in melancholic, psychotic, retarded, medically burdened, or inflammatory-metabolic depressive phenotypes. Yet a single cortisol, prolactin, CRP, IL-6, or BDNF value is unlikely to define the therapeutic mechanism. The Postictal Systems Window Hypothesis treats these markers as timed curves and coupling signals. Their meaning depends on whether they rise, peak, recover, and coordinate with EEG suppression and recovery, hemodynamic-metabolic transition, reorientation, and early clinical change.

The distinctive claim of the Postictal Systems Window Hypothesis is therefore not that ECT affects networks, hormones, inflammation, perfusion, glia, or plasticity. Those observations are already established to varying degrees. The distinctive claim is that an adequate seizure opens a transient, repeated, biologically organized postictal window whose amplitude, coordination, anatomical distribution, and duration determine whether the treatment becomes therapeutic, ineffective, or cognitively toxic. This hypothesis predicts that early postictal curves after the first one to three treatments should outperform seizure duration, isolated network measures, isolated endocrine or inflammatory markers, and late structural plasticity markers in predicting remission, cognitive burden, and the need for technique adjustment.

This distinction also clarifies several clinical observations that are incompletely explained by stand-alone models: similar seizure durations may lead to different outcomes; technically adequate seizures may fail when postictal recovery is weak or uncoupled; bilateral, unilateral, brief-pulse, ultrabrief-pulse, anesthetic, and dosing differences may alter both efficacy and cognition without simply changing whether a seizure occurred; and cognitive toxicity may reflect an excessive, prolonged, or anatomically unfavorable window rather than being inseparable from antidepressant efficacy.

### 1.4 Scope and literature selection

This article is a conceptual review and mechanistic perspective for a broad psychiatric readership. It focuses on ECT in major depressive disorder, especially severe, melancholic, psychotic, suicidal, catatonic or retarded, and treatment-resistant presentations (1, 3, 9, 10). Bipolar depression and catatonia outside depression are clinically related but are not the central focus.

The review is narrative rather than systematic. We prioritized clinical ECT studies, systematic reviews and meta-analyses, human mechanistic studies, major neuroimaging and biomarker papers, and selected animal studies where human data are sparse. The purpose is not to provide an exhaustive catalogue of ECT biology, but to ask whether existing findings can be ordered into a clinically testable temporal model. Where evidence is weakest — particularly BBB/NVU permeability and some glial mechanisms in humans — the text states this explicitly.

## 2 Clinical phenotype of ECT response in depression

The strongest reason to seek a systems-level mechanism is the clinical phenotype of ECT response (1, 13). ECT is typically considered when depression is severe, dangerous, psychotic, melancholic, catatonic or profoundly retarded, medically compromising, suicidal, or resistant to adequate pharmacological and psychological treatment (1, 3, 9, 10). In such states, depression is not expressed only as low mood. It may involve psychomotor arrest or agitation, delusional guilt or nihilism, inability to eat or sleep, autonomic and endocrine dysregulation, cognitive fixation, and imminent risk to life (10–12, 25–27). A treatment capable of changing this clinical state over days to weeks is unlikely to be fully explained by a narrow monoaminergic or single-pathway mechanism (1, 13).

Several clinical observations motivate the proposed framework without, by themselves, proving it. Large ECT cohorts and meta-analytic work support substantial response and remission rates, with particularly strong outcomes reported in severe, psychotic, melancholic, and older-age depressive presentations, although prediction for an individual patient remains imprecise (3, 28–31). Psychomotor improvement has also been linked to decreased IL-6 during ECT (32). Suicidal ideation may decrease before full syndromal remission, suggesting early changes in salience, arousal, psychomotor drive, sleep pressure, and the capacity to disengage from catastrophic self-referential belief (33–35). Response is also cumulative: some patients improve after one or two treatments, whereas many require a full course, and relapse remains common without continuation pharmacotherapy or maintenance ECT (1, 28, 36–40).

Cognitive adverse effects are part of the same clinical phenotype and must be included in any credible mechanism. ECT can produce acute disorientation, impaired new learning during the course, and retrograde autobiographical memory loss (1, 3). Objective cognitive performance often worsens acutely and may recover after treatment, but standardized tests do not fully capture autobiographical memory burden or patient experience (41, 42). Pulse width, electrode placement, stimulus dosing, number of treatments, age, medication exposure, and individual vulnerability all influence cognitive risk (1, 43–45). A clinically useful mechanism must therefore explain not only why ECT can work rapidly and cumulatively, but also why efficacy and cognitive toxicity may be coupled in some patients and partly dissociated in others (1, 44). These clinical observations provide the rationale for a temporally ordered model, but their mechanistic interpretation is developed after the hierarchy of the postictal systems window has been defined.

## 3 Existing mechanistic accounts and their place in the hierarchy

Existing models provide important pieces of the mechanism. Their limitation is not falsity, but insufficient temporal and causal positioning (4, 13). **Table 1** defines the hierarchy used in this manuscript. **Table 2** summarizes the major mechanistic accounts and how each is incorporated without allowing the model to become a synonym for all postictal biology.

**Table 1:** Hierarchical architecture of the postictal systems window hypothesis.

Level	Domain	Role in the model	Candidate operational markers	Hierarchical rationale
1	Therapeutic seizure induction	Necessary trigger; not sufficient mediator	Seizure threshold; stimulus dose relative to threshold; ictal morphology and amplitude; motor and EEG seizure duration; seizure-quality indices	Conventional ECT requires seizure induction, but seizure duration alone is an insufficient proxy for therapeutic engagement. The clinically relevant event is proposed to emerge from the postictal state rather than from the seizure itself.
2A	Postictal network interruption and recovery	Core electrophysiological-clinical mediator	Postictal suppression index; EEG recovery slope; persistence of postictal slowing; time to reorientation; early restoration of self, place, and time orientation	This is the most accessible routine signal of the immediate postictal transition. The model predicts that recovery quality should carry more mechanistic and prognostic information than seizure duration alone.
2B	Hemodynamic-metabolic transition	Core physiological bridge component	Arterial spin labeling perfusion; diffuse optical/NIRS monitoring; oxygenation and perfusion recovery curves; lactate where feasible	Seizures impose major metabolic demand. Structured postictal perfusion and oxygenation dynamics may determine whether network recovery is coordinated, contained, and biologically permissive for clinical reorganization.
3	HPA-axis and immune-metabolic coupling	Coupled modulators and stratification signals	Cortisol, ACTH, and prolactin trajectories; CRP and IL-6; selected kynurenine-pathway metabolites; pre-treatment, early postictal, 2-hour, and 24-hour sampling	Severe depressive phenotypes often involve stress-system, inflammatory, and metabolic dysregulation. Dynamic coupling across endocrine, immune, EEG, and clinical recovery domains is expected to be more informative than isolated baseline values.
4	Glial, synaptic, trophic, structural, and epigenetic change	Translation and consolidation mechanisms	BDNF and VEGF; hippocampal and network imaging; methylation signatures; microRNAs; longer-term cognitive and remission trajectories	These processes plausibly explain durability, cumulative course effects, relapse vulnerability, and memory-network risk. They are positioned downstream because they are unlikely to explain immediate clinical reorientation alone.
5	BBB/NVU and perivascular exchange	Second-order exploratory interface	DCE-MRI; selected serum/CSF markers such as S100B, GFAP, neurofilament light chain, and albumin quotient, only where clinically justified	Current human evidence does not establish therapeutic BBB opening during routine ECT. This domain remains biologically plausible and testable, but it is not required for the core hypothesis.

ACTH, adrenocorticotropic hormone; ASL, arterial spin labeling; BBB, blood-brain barrier; BDNF, brain-derived neurotrophic factor; CRP, C-reactive protein; CSF, cerebrospinal fluid; DCE-MRI, dynamic contrast-enhanced magnetic resonance imaging; EEG, electroencephalography; ECT, electroconvulsive therapy; GFAP, glial fibrillary acidic protein; HPA, hypothalamic-pituitary-adrenal; IL-6, interleukin-6; NIRS, near-infrared spectroscopy; NVU, neurovascular unit; VEGF, vascular endothelial growth factor.

**Table 2:** Existing mechanistic accounts of ECT and their placement within the postictal systems window hypothesis.

Mechanistic account	Representative evidence	Explanatory value	Why insufficient as a stand-alone model	Placement in the postictal-window hierarchy
Monoaminergic and receptor adaptation	ECT is associated with changes in serotonergic, noradrenergic, dopaminergic, cholinergic and receptor-level signaling.	Links ECT to established antidepressant pharmacology and to cumulative symptomatic adaptation.	Does not account for rapid postictal recovery, high efficacy in psychotic or melancholic states, hemodynamic-endocrine coupling, or technique-sensitive cognitive effects.	Downstream modulator: contributes to symptomatic consolidation within a broader postictal sequence.
Glutamate-GABA and excitatory-inhibitory balance	Seizure induction necessarily recruits glutamatergic excitation, inhibitory rebound and postictal suppression; MRS studies report ECT-associated GABA changes.	Explains seizure adequacy, postictal inhibition, and the relevance of anticonvulsants, anesthetic choice and seizure threshold.	Too narrow to explain endocrine, immune-metabolic, neurovascular, glial and cognitive recovery trajectories.	Proximal neural stabilizer: shapes the amplitude and duration of the core window.
HPA-axis and endocrine response	ECT acutely alters cortisol, ACTH, prolactin and related endocrine markers; severe depression often involves HPA-axis dysregulation.	Connects melancholia, neurovegetative symptoms, stress-system flexibility and systemic engagement.	Single hormone values are nonspecific and do not define network interruption, hemodynamic recovery or cognitive risk.	Coupled systemic curve: most informative when timed against EEG recovery, reorientation and clinical trajectory.
Immune-inflammatory and kynurenine modulation	CRP, IL-6, other cytokines and kynurenine-pathway metabolites vary by baseline inflammatory state, timing and clinical response.	Supports stratification of inflammatory-metabolic depressive phenotypes and may explain psychomotor, fatigue and anhedonic components.	Peripheral markers may not reflect central immune states; acute inflammatory activation may indicate engagement rather than injury.	Dynamic modulator and stratification signal: not yet a validated clinical test.
Network disruption and functional rewiring	Functional imaging and EEG studies show changes in frontal, default-mode, salience, cognitive-control and limbic network organization after ECT.	Explains interruption of rigid self-reference, suicidal fixation, psychotic certainty, salience distortion and psychomotor arrest.	Becomes too abstract if separated from postictal recovery, hemodynamic-metabolic physiology and systemic biological engagement.	Core network expression: the circuit-level manifestation of the postictal transition.
Neurovascular coupling and hemodynamic transition	ECT seizures are followed by perfusion and oxygenation changes; recent optical data describe hyperemic waves consistent with cortical spreading depolarization.	Shows that the postictal period contains organized physiology after seizure termination and links neural recovery to metabolic support.	Hemodynamic change alone does not prove antidepressant causality or define the optimal therapeutic window.	Core bridge component: connects seizure expression, network recovery and systemic physiology.
Glial biology	Preclinical studies show astrocytic and microglial responses after electroconvulsive stimulation, including changes relevant to vascular endfeet, purinergic signaling and synaptic remodeling.	Provides cellular translators between neurovascular, immune, metabolic and synaptic processes.	Direct human evidence remains limited; glial activation is not automatically therapeutic.	Translator mechanism: links the core window to downstream remodeling and consolidation.
Trophic, structural and epigenetic plasticity	ECT is associated with BDNF, VEGF, hippocampal and amygdala volume changes, methylation signatures and microRNA signals.	Explains cumulative course effects, durability of response, relapse vulnerability and longer-term biological remodeling.	Too slow and nonspecific to explain immediate reorientation or early reductions in suicidal, psychotic or psychomotor symptoms.	Downstream consolidation: repeated windows are translated into more durable change.
Hippocampal and autobiographical memory-network exposure	Imaging and electric-field studies link medial temporal exposure, hippocampal change and memory-network involvement to cognitive outcomes.	Clarifies why antidepressant efficacy and cognitive burden may be coupled in some patients and partly dissociated in others.	Does not by itself explain remission; hippocampal volume change is not equivalent to antidepressant response.	Anatomical-distribution and cognitive-risk pathway: central to technique optimization and memory-sparing strategies.
BBB/NVU and perivascular exchange	Animal evidence for transient permeability or astrovascular change is limited; sparse human evidence does not support gross therapeutic BBB opening during routine ECT.	Offers a plausible brain-body interface for endothelial signaling, cytokine communication, metabolite flux and perivascular exchange.	Therapeutic BBB opening is not established in humans and is not required for the central hypothesis.	Second-order exploratory interface: biologically plausible, testable, but not a core mediator.

Note. Placement indicates proposed mechanistic rank within the postictal systems window hypothesis, not proof of causality. The table is intended to prevent causally flat interpretation of ECT biology. Abbreviations: ACTH, adrenocorticotropic hormone; BBB, blood-brain barrier; BDNF, brain-derived neurotrophic factor; CRP, C-reactive protein; CSD, cortical spreading depolarization; ECT, electroconvulsive therapy; EEG, electroencephalography; GABA, gamma-aminobutyric acid; HPA, hypothalamic-pituitary-adrenal; IL-6, interleukin-6; MRS, magnetic resonance spectroscopy; NVU, neurovascular unit; VEGF, vascular endothelial growth factor.

### 3.1 Neurotransmitter release and receptor adaptation

ECT alters serotonergic, noradrenergic, dopaminergic, and cholinergic systems, and early theories often interpreted antidepressant action through acute release and receptor adaptation (46–48). Monoaminergic systems regulate mood, salience, sleep, appetite, psychomotor activation, and cognition (46, 49). These effects remain clinically plausible but are better understood as downstream modulation within a broader postictal sequence (4, 50). Monoamine change alone does not explain rapid shifts in psychotic or melancholic depression, technique-sensitive cognitive burden, or the importance of postictal recovery (4, 13).

### 3.2 Glutamate, GABA, and excitatory-inhibitory balance

A seizure necessarily recruits excitatory and inhibitory systems (50, 51). Magnetic resonance spectroscopy studies have reported changes in cortical GABA during ECT for depression (8, 9). Glutamatergic signaling is implicated by seizure induction, postictal inhibition, synaptic plasticity, and the wider literature on rapid antidepressant effects (52–54). Excitatory-inhibitory mechanisms are close to the core because they shape seizure expression and postictal suppression (51, 55). They do not, by themselves, explain endocrine, vascular, immune, metabolic, and cognitive recovery profiles (4, 13).

### 3.3 Neuroendocrine effects and the HPA axis

The HPA axis is central to severe depression (56, 57). Large cohort data demonstrate altered HPA-axis activity in major depressive disorder, particularly in more severe presentations (57). ECT acutely changes cortisol, adrenocorticotropic hormone, prolactin, and related endocrine markers (56, 58). Some studies suggest HPA-axis normalization after ECT, including in medication-resistant patients (1). Stimulus intensity has been associated with prolactin and cortisol release during unilateral ECT (56, 58). Post-dexamethasone cortisol has been examined as a predictor of response (56, 57). These endocrine findings should be interpreted cautiously because acute hormonal release may reflect nonspecific procedural stress, seizure physiology, anesthetic effects, circadian timing, or baseline melancholic biology rather than a direct antidepressant mediator.

The postictal systems window hypothesis does not treat a single cortisol value as a mechanism. It treats endocrine dynamics as a timed curve: baseline, rise, peak, recovery, and coupling to EEG, autonomic, inflammatory, and clinical trajectories (56, 57). In melancholic and psychotic depression, this systemic curve may be particularly informative because endocrine and neurovegetative signs are often clinically prominent (57).

### 3.4 Neurotrophic, structural, and epigenetic plasticity

ECT is associated with changes in BDNF, VEGF, and other plasticity-related mediators (59–62). Neuroimaging studies repeatedly find ECT-associated increases in hippocampal and amygdala volume (13, 61). Large multisite studies confirm hippocampal change while showing that volume increase is not simply identical with antidepressant response (63, 64). ECT-associated hippocampal or amygdala volume change is biologically important but should not be equated with clinical response. Structural change may be a correlate, a downstream consolidation signal, or in some patients a marker of memory-network exposure. Human methylome-wide, stress-gene, and microRNA studies suggest treatment- and response-associated signals (65–68).

These findings provide a credible bridge from brief repeated treatments to durable clinical change. They are placed downstream in the present model (4, 13). Plasticity is not intrinsically therapeutic. It may consolidate recovery, remain clinically inert, or contribute to cognitive adverse effects depending on timing, anatomical distribution, and individual vulnerability (61, 63). The hippocampal signal is particularly important because it may sit at the boundary between therapeutic plasticity and memory-network risk (63, 64, 68).

### 3.5 Immune, inflammatory, and kynurenine-pathway modulation

Depression is immunologically heterogeneous (69, 70). Some patients show elevated C-reactive protein (CRP), interleukin-6 (IL-6), tumor necrosis factor-alpha, interleukin-8, altered interleukin-10, oxidative stress, or changes in tryptophan-kynurenine metabolism (69–71). ECT interacts with this biology. Systematic reviews describe acute and course-related inflammatory changes, with substantial variation by marker, timing, medication exposure, assay, and subgroup (70, 72). A recent meta-analysis reported that higher baseline CRP and IL-6 were associated with greater symptom improvement after ECT and that changes in kynurenine metabolites and IL-8 were associated with improvement, while emphasizing the need for standardized prospective work (69). The inflammatory literature remains heterogeneous, and peripheral cytokine or CRP changes should not be overread as direct evidence of central immune mediation. Their strongest current value is as possible subgroup or dynamic-coupling markers.

Kynurenine-pathway studies are relevant because they connect immune tone, tryptophan metabolism, glutamatergic modulation, oxidative stress, fatigue, anhedonia, and cognitive slowing (69, 71). The model treats these markers as coupled modulators and stratification signals, not as validated clinical tests (69, 70). Peripheral inflammatory markers cannot be assumed to represent central immune states (70). Acute inflammatory activation after ECT may represent repair signaling or physiological engagement rather than harm (70, 72).

### 3.6 Network disruption and functional rewiring

Network models are among the most clinically persuasive accounts of ECT (13, 73). Severe depression is often characterized by rigid self-referential cognition, impaired cognitive control, disturbed salience processing, limbic overconstraint, and psychomotor inhibition (13, 74). ECT has been associated with reduced frontal cortical connectivity in severe depression. and with reorganization of intra-network and inter-network connectivity (73–77). Contemporary magnetic resonance reviews describe ECT as producing disruption, plasticity, and subsequent rewiring (13, 78).

Network interruption is close to the core of the hypothesis because it maps well onto the clinical release of psychotic guilt, nihilistic certainty, psychomotor arrest, agitation, or suicidal fixation (74). Its limitation is that functional connectivity alone can become too abstract. It does not explain why endocrine output, hemodynamic physiology, inflammatory tone, glial behavior, and reorientation are engaged in the same treatment session (4, 13).

### 3.7 Neurovascular coupling, BBB, and NVU effects

The neurovascular unit comprises endothelial cells, pericytes, astrocytic endfeet, basement membrane, neurons, microglia, and extracellular matrix. It regulates perfusion, metabolic exchange, immune signaling, and BBB integrity (79). Modern BBB biology emphasizes a dynamic interface rather than a static wall (80). The distinction between neurovascular coupling and BBB permeability is crucial for this manuscript.

Neurovascular coupling and hemodynamic response belong near the core because seizures impose major metabolic demands and postictal recovery occurs under changing cerebral blood flow and oxygenation (13, 50). By contrast, BBB/NVU permeability is retained as an exploratory interface. Existing evidence does not establish gross therapeutic BBB opening during routine human ECT, although preclinical electroconvulsive stimulation studies justify testing whether selective, brief, reversible interface changes can be detected with modern methods and whether they relate to response or cognitive burden (68, 79–82).

## 4 The postictal systems window hypothesis

### 4.1 Definition

The postictal systems window is defined as a transient, repeated, coordinated biological state after each adequate ECT-induced seizure. It is not synonymous with seizure duration, postictal confusion, or postictal EEG suppression. It is a time-limited state during which depressive allostasis may become destabilized and more susceptible to reorganization.

In mechanistic terms, the window refers to the phase of postictal recoupling rather than to any single marker. EEG suppression and recovery, reorientation, neurovascular-metabolic transition, endocrine activation, and immune-metabolic signaling are interpreted as temporally related readouts of the same recovery process, not as independent mechanisms of equal rank.

**Figure 1:**
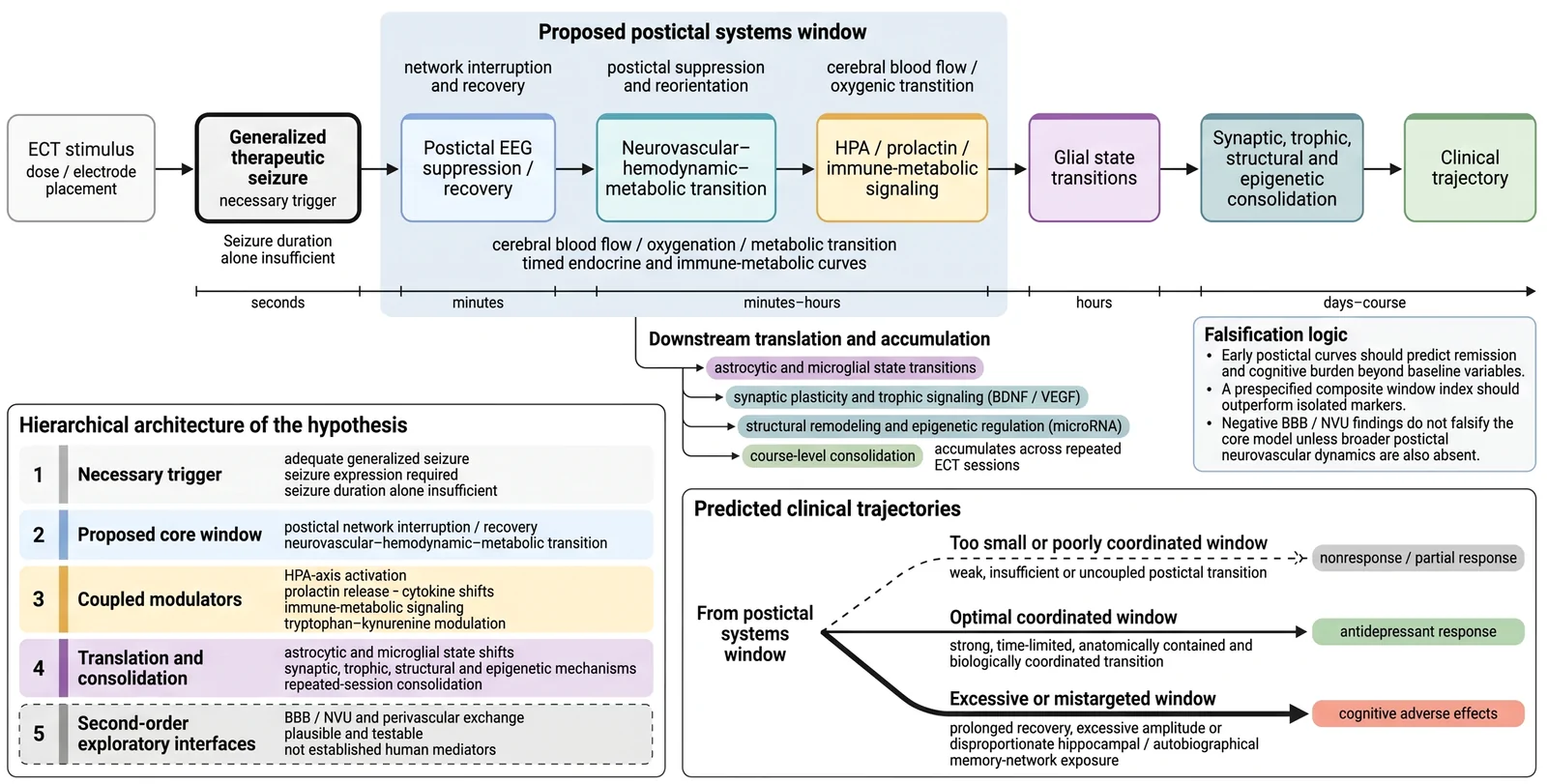
Expanded conceptual model of the postictal systems window hypothesis. An adequate ECT-induced seizure opens a transient postictal period in which EEG suppression and recovery, network interruption and reorientation, and the neurovascular-hemodynamic-metabolic transition form the proposed core window and become coupled to HPA-axis and immune-metabolic signaling. Glial, synaptic, trophic, structural, and epigenetic processes are positioned mainly downstream as translation and consolidation mechanisms, whereas BBB/NVU permeability and perivascular exchange remain second-order exploratory interfaces. The model predicts three clinically relevant trajectories: too small or uncoupled, optimal and contained, or excessive and anatomically unfavorable, associated with nonresponse, antidepressant response, or cognitive adverse effects, respectively. BBB, blood–brain barrier; ECT, electroconvulsive therapy; EEG, electroencephalography; HPA, hypothalamic–pituitary–adrenal; NVU, neurovascular unit.

The four dimensions introduced above can be defined more formally as follows. Amplitude refers to the strength of the perturbation. Coordination refers to coupling among EEG suppression and recovery, hemodynamic response, endocrine pulsatility, immune-metabolic signaling, and cognitive reorientation. Anatomical distribution refers to which networks and structures are exposed. Duration refers to how quickly the system returns to baseline or enters a new equilibrium. **Figure 1** depicts the expanded conceptual model and the three clinically relevant trajectories: too small or uncoupled, optimal and contained, or excessive and anatomically unfavorable.

### 4.2 Hierarchical architecture: what is core, what is coupled, and what remains exploratory

**Figure 2:**
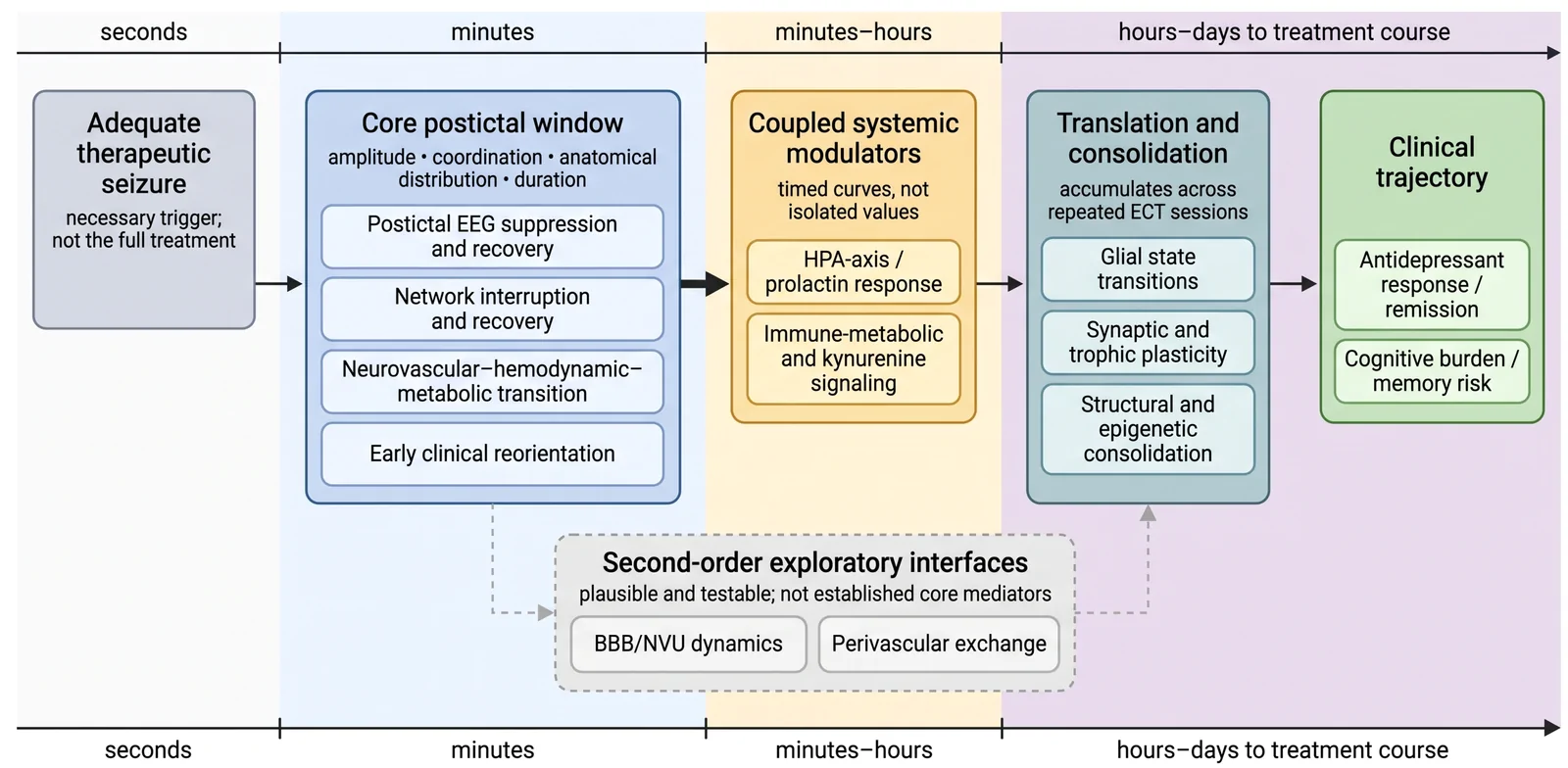
Temporal and hierarchical sequence of the postictal systems window hypothesis. The therapeutic seizure acts as the necessary trigger, followed over minutes by the proposed core postictal window, including EEG suppression and recovery, network interruption and recovery, neurovascular-hemodynamic-metabolic transition, and early clinical reorientation. Over minutes to hours, HPA-axis and immune-metabolic/kynurenine signals act as coupled systemic modulators. Repeated ECT sessions are then translated over hours to days and across the treatment course into glial, synaptic, trophic, structural, and epigenetic consolidation, culminating in antidepressant response and/or cognitive burden. BBB/NVU dynamics and perivascular exchange are shown as second-order exploratory interfaces rather than established mediators. BBB, blood–brain barrier; ECT, electroconvulsive therapy; EEG, electroencephalography; HPA, hypothalamic–pituitary–adrenal; NVU, neurovascular unit.

The model is intentionally integrative, but it is not causally flat. At the first level is the seizure, which is necessary for conventional ECT but not by itself the full treatment (14, 83). At the second level is the proposed core postictal event: network interruption and recovery, early reorientation, and a hemodynamic-metabolic transition (84–86). At the third level are coupled systemic modulators: HPA-axis and immune-metabolic curves that may identify whether the organism has entered a coordinated state (57, 69). At the fourth level are consolidation mechanisms: synaptic, trophic, structural, and epigenetic changes that make repeated sessions durable (59, 61, 65). At the fifth level are second-order interfaces such as BBB/NVU permeability and perivascular exchange, which may be relevant but cannot be treated as established mediators in humans (87). **Figure 2** provides a concise schematic summary of this hierarchical architecture and its proposed temporal sequence, from the ictal trigger to the core postictal window, coupled systemic modulation, downstream consolidation, and exploratory second-order interfaces.

This architecture generates a clear falsification logic. The hypothesis does not require every marker to change in every responder. It requires that early, coordinated postictal dynamics carry more predictive and mechanistic information than isolated baseline variables or seizure duration alone. It also predicts that technique, anesthesia, age, vascular burden, and medications shape the window rather than merely determining whether a seizure occurred (18, 83).

Because the model combines domains with unequal evidentiary strength, the postictal systems window should be understood as a testable construct rather than as an established empirical entity. Seizure induction, postictal EEG suppression and recovery, time to reorientation, endocrine activation, and ECT-associated network or structural changes are supported by direct or relatively consistent human ECT evidence. Inflammatory-metabolic signaling and neurovascular-hemodynamic dynamics are supported by convergent but more heterogeneous or associative evidence. Glial translation, BBB/NVU permeability, and perivascular exchange remain exploratory, with mainly preclinical or indirect human support. **Table 3** summarizes this evidence hierarchy and distinguishes established ECT biology from components that remain inferential or exploratory. The central claim is not that a coordinated systems window has already been demonstrated, but that it can be tested prospectively: early multimodal postictal dynamics should predict remission and cognitive burden beyond conventional clinical and procedural variables. Failure to demonstrate such predictive value would weaken the hypothesis.

**Table 3:** Evidence status of components included in the postictal systems window hypothesis.

Component	Evidence status	What current evidence reasonably supports	What remains unproven and why it matters
Therapeutic seizure induction	Strong procedural evidence	An adequate ECT-induced seizure is required for conventional ECT and is shaped by electrode placement, stimulus dose, pulse width, anesthetic agent, medications, and seizure threshold.	Seizure occurrence or duration alone does not adequately explain remission, nonresponse, or cognitive toxicity. In the model, the seizure is the necessary trigger, not the full therapeutic event.
Postictal EEG suppression and recovery	Moderate direct human evidence	Postictal suppression, EEG recovery slope, postictal slowing, and seizure quality provide more information than motor seizure duration alone. Several studies link postictal suppression or recovery to clinical outcome or reorientation.	No single EEG marker is a validated stand-alone predictor of antidepressant response or cognitive burden. These measures are treated as accessible signals of the early postictal window.
Time to reorientation and early clinical recovery	Moderate direct human evidence	Reorientation time is clinically observable, patient-relevant, and related to postictal recovery and cognitive burden in human ECT studies.	It remains unclear whether delayed reorientation is a mediator of cognitive risk, a safety marker, or a nonspecific recovery marker. In the model, it indexes window duration and containment.
Network interruption and functional reorganization	Moderate to strong human evidence for ECT-associated change; weaker evidence for mediation	ECT is associated with changes in frontal, limbic, default-mode, salience, cognitive-control, and psychomotor networks. These changes plausibly relate to loosening of depressive rigidity, suicidal fixation, psychotic preoccupation, and psychomotor retardation.	Network change alone does not define the timing, systemic coupling, or cognitive-risk profile of the postictal state. In the model, it is the circuit-level expression of the window, not the whole mechanism.
Neurovascular and hemodynamic transition	Emerging human and translational evidence	Seizures impose major metabolic demand, and human imaging studies support postictal perfusion and hemodynamic changes. Recent optical data suggest that the postictal period may contain organized wave-like physiology rather than simple seizure termination.	A causal link between these hemodynamic events and antidepressant response remains unproven. The model treats them as a physiological bridge between seizure expression, network recovery, and systemic biology.
HPA-axis and endocrine activation	Moderate human evidence for acute ECT-related hormonal responses; weaker evidence for prediction	ECT acutely alters cortisol, ACTH, prolactin, and related endocrine markers. This is particularly relevant in melancholic, psychotic, retarded, or neurovegetative depressive phenotypes.	A single hormone value is unlikely to define mechanism. The relevant test is whether timed endocrine curves coordinate with EEG recovery, reorientation, and clinical change.
Immune-inflammatory and kynurenine-pathway changes	Moderate associative human evidence; heterogeneous findings	Baseline inflammatory status and changes in CRP, IL-6, cytokines, and kynurenine-pathway metabolites may relate to ECT response in biologically defined subgroups.	Peripheral markers may not reflect central immune states. Acute inflammatory activation may represent physiological engagement rather than injury or direct therapeutic mediation.
Trophic, structural, and epigenetic plasticity	Moderate evidence for ECT-associated change; limited evidence for direct causality	ECT is associated with changes in BDNF, VEGF, hippocampal and amygdala volume, methylation signatures, and microRNAs. These mechanisms plausibly contribute to cumulative and durable response.	These processes are unlikely to fully explain immediate postictal reorientation or early clinical improvement after one or a few treatments. In the model, they are downstream consolidation mechanisms.
Glial translation	Low direct human evidence; mainly preclinical and mechanistic support with substantial translational limitations	Whether animal BBB/NVU findings translate to selective, brief, clinically meaningful BBB/NVU changes during routine human ECT remains unproven.	Human evidence remains limited, and glial activation cannot be assumed to be therapeutic. This component remains exploratory.
BBB/NVU and perivascular exchange	Low to very low evidence as a human ECT mediator	Animal studies suggest possible transient BBB/NVU or astrovascular effects after electroconvulsive stimulation. Human data do not establish gross therapeutic BBB opening during routine ECT.	Selective, brief, clinically meaningful BBB/NVU changes remain speculative. This is a second-order exploratory interface, not a required component of the core hypothesis.
Cognitive burden as excessive, prolonged, or anatomically unfavorable window exposure	Moderate associative human evidence; mechanistic interpretation remains inferential	Cognitive effects vary by electrode placement, pulse width, stimulus dose, treatment number, age, medication exposure, reorientation time, and medial temporal or hippocampal exposure.	It remains unproven that cognitive toxicity is caused by an excessive or mistargeted postictal window. This component is central for safety testing and falsification of the model.
Integrated postictal systems window	Conceptual; not yet demonstrated as a unified construct	The framework links seizure induction, postictal recovery, hemodynamic-metabolic transition, systemic modulation, and downstream consolidation into a temporal hierarchy.	No prospective multimodal study has yet shown that these components form a coordinated window predicting remission and cognitive burden better than isolated markers. This is the central hypothesis to be tested.

Note. Evidence status refers to the directness and consistency of evidence supporting each component as part of ECT biology; it is not a formal GRADE rating. The table distinguishes evidence-supported components from inferential or exploratory elements of the Postictal Systems Window Hypothesis. The integrated window itself should be interpreted as a prospective, falsifiable construct rather than as an established empirical entity. Abbreviations: ACTH, adrenocorticotropic hormone; BBB, blood-brain barrier; BDNF, brain-derived neurotrophic factor; CRP, C-reactive protein; ECT, electroconvulsive therapy; EEG, electroencephalography; HPA, hypothalamic-pituitary-adrenal; IL-6, interleukin-6; NVU, neurovascular unit; VEGF, vascular endothelial growth factor.

### 4.3 The seizure as trigger, not the whole treatment

A seizure initiates ictal excitation, recruitment of inhibitory mechanisms, postictal suppression, autonomic and endocrine activation, changes in perfusion and metabolism, inflammatory and glial signaling, and recovery (83, 88, 89). The clinical question is therefore not merely whether a seizure occurred or how long it lasted, but what kind of postictal state followed. A patient can have an apparently adequate seizure and remain depressed. Another can improve but experience unacceptable cognitive burden. Bilateral and unilateral placements, brief and ultrabrief pulse widths, anesthetic agents, stimulus dosing, and concomitant medications can produce different clinical profiles despite all producing seizures (83). The postictal systems window is the candidate mediator that links technique to outcome.

### 4.4 Postictal suppression, recovery slope, and reorientation as core clinical signals

Postictal EEG suppression is the most accessible marker of the immediate postictal state (14, 18). Studies have linked seizure expression and postictal suppression to clinical outcome and cognitive effects, although findings have not been uniform (83). Postictal suppression has also been reported to correlate with therapeutic efficacy in bilateral ECT (14). However, postictal suppression should not be interpreted as a validated stand-alone biomarker of response. In the present model, its value depends on whether it improves prediction when combined with recovery slope, reorientation, hemodynamic-metabolic dynamics, and clinical trajectory. The present model does not treat postictal suppression as a sufficient biomarker. It treats it as an electrophysiological sign that a larger transition may have occurred.

Recovery slope and time to reorientation are equally important because they place EEG findings into a clinically visible trajectory (90). Severe depression can be understood as a self-stabilizing state in which guilt, threat, insomnia, psychomotor inhibition, autonomic arousal, and suicidal inevitability reinforce one another. A sufficient postictal interruption may loosen this pathological constraint. A prolonged or delirious recovery may signal that the window is excessive or poorly contained, particularly in older or medically vulnerable patients (15).

### 4.5 Neurovascular and hemodynamic waves: a strategic anchor, not a complete mechanism

Seizures impose large metabolic demands and produce marked changes in cerebral blood flow (16). The postictal period is therefore not simply electrical silence. It is a neurovascular transition. Recent optical neuroimaging work reported that ECT generates a postictal wave of spreading depolarization in mice and humans, with human hyperemic waves consistent with that process and shaped by stimulation parameters (86). The key implication is not that cortical spreading depolarization alone explains antidepressant response. The key implication is that the postictal period contains a structured second event after the seizure and that stimulation parameters can shape its dynamics.

This observation is a strategic anchor for the postictal systems window hypothesis. It supports the central idea that what follows the seizure may be biologically organized and clinically relevant. A neurovascular-hemodynamic transition could matter in depression because mood, psychomotor function, sleep, salience, and cognition depend on coupling between neuronal activity, blood flow, metabolic support, astrocytic function, and vascular reactivity. The model does not require vascular injury. It requires a controlled hemodynamic and metabolic transition in which circuits recover under altered neurovascular conditions.

### 4.6 BBB and NVU dynamics as a second-order interface

BBB/NVU hypotheses have become prominent in ECT mechanism discussions because ECT combines a generalized seizure, abrupt autonomic and hemodynamic shifts, increased metabolic demand, postictal recovery, and clinically visible cognitive effects. This makes the neurovascular unit an attractive candidate interface between seizure physiology, brain perfusion, systemic signaling, and postictal clinical outcome (79–81).

In the present framework, however, BBB/NVU dynamics are treated as a second-order exploratory interface rather than as a central mediator. The model does not require crude or gross therapeutic BBB opening. If relevant BBB/NVU changes occur in humans, they would more plausibly involve selective, brief, and reversible processes such as altered transport, endothelial signaling, perivascular exchange, cytokine-related communication, endocrine access, metabolite flux, or repair-associated signaling (87, 91). Current evidence justifies testing this possibility, not assuming it.

The translational boundary remains important. Much of the supportive BBB/NVU evidence comes from preclinical electroconvulsive stimulation paradigms, which differ from clinical ECT in species, brain scale, vascular architecture, stimulation parameters, anesthesia, seizure expression, and timing of tissue assessment. Human studies rely largely on indirect serum markers, imaging proxies, or selected clinical samples, each with limited specificity. Positive animal findings should therefore be considered hypothesis-generating rather than confirmatory, and the absence of gross BBB opening in human studies should not be ignored (79–81).

This distinction matters for falsification. A negative DCE-MRI or serum-marker study showing no gross BBB opening would weaken the BBB/NVU interface component, but would not falsify the core postictal systems window hypothesis. In contrast, repeated failure of early EEG recovery, reorientation, hemodynamic-metabolic dynamics, and systemic curves to predict clinical or cognitive outcomes would challenge the model more directly.

### 4.7 HPA-axis pulsatility and immune-metabolic recoding

ECT-induced hormonal release is often described as nonspecific stress physiology. That is partly true and partly insufficient (57). Severe depression frequently involves loss of flexibility in sleep, circadian timing, appetite, stress response, psychomotor activation, and autonomic tone. A controlled endocrine challenge may reveal whether the HPA axis can mount, terminate, and recalibrate a response. The clinically meaningful pattern may be a brisk and contained response coupled to postictal suppression and subsequent recovery. A prolonged, blunted, or poorly coordinated endocrine response may carry a different meaning.

Inflammation in depression is not a single state (69, 70). High CRP, IL-6, other cytokines, oxidative stress, kynurenine-pathway activation, and metabolic disturbance may appear in different combinations. ECT may acutely activate some immune markers while, across a course, modifying the relationship among inflammation, metabolism, stress physiology, and depressive symptoms (69, 72). We use the term immune-metabolic recoding to describe this proposed change in pattern, timing, recovery, and clinical coupling of immune-metabolic responses, not a simple transient rise or fall in inflammatory markers.

This distinction is important. Transient immune activation refers to short-lived post-treatment changes in markers such as CRP, IL-6, cytokines, lactate, or kynurenine-pathway metabolites. Immune-metabolic recoding, by contrast, would require evidence that repeated postictal states alter the patient’s immune-metabolic response profile over time: lower or more flexible baseline inflammatory tone before later sessions, faster resolution after acute activation, altered kynurenine-pathway balance, reduced coupling between inflammation and psychomotor or anhedonic symptoms, or closer temporal alignment between immune-metabolic trajectories and clinical improvement. Such recoding should therefore be tested with longitudinal curves, not inferred from a single post-ECT blood sample.

Patients with higher baseline inflammatory signals may respond particularly well because their depressive state is partly maintained by immune-metabolic coupling (69). This remains a stratification hypothesis rather than a routine clinical biomarker. The model predicts that immune-metabolic measures will be most informative when interpreted alongside EEG recovery, reorientation, endocrine dynamics, sleep, psychomotor change, and symptom trajectory, rather than as isolated inflammatory values.

### 4.8 Glia as translators and consolidation as the durable endpoint

In this framework, astrocytes and microglia are best positioned as potential translators of postictal physiology into synaptic, metabolic, vascular, and immune-related change. Astrocytic endfeet regulate neurovascular coupling and BBB function (87, 91). Astrocytes handle glutamate, buffer extracellular potassium, support lactate metabolism, influence aquaporin-4-dependent water movement, and participate in cytokine signaling (91, 92). Microglia survey synapses, respond to purinergic signaling, shape inflammatory tone, and contribute to pruning and plasticity (93, 94). Electroconvulsive seizure models show glial activation (95). Experimental work has reported enhanced microglial motility and purinergic currents after electroconvulsive shock in the mouse hippocampus (93). Animal data also suggest that electroconvulsive shock may restore astrocytic endfoot coverage of brain blood vessels in models of depressive-like behavior (87). These findings are not direct proof of the human antidepressant mechanism. They make glia plausible translators of the postictal state rather than established mediators of clinical response (94–96).

The translational limitations are substantial. Most glial evidence relevant to electroconvulsive interventions comes from animal models using electroconvulsive stimulation rather than clinical ECT. Such models allow direct assessment of astrocytes, microglia, vascular endfeet, purinergic signaling, and synaptic remodeling, but they do not reproduce the full clinical context of human depression, anesthesia, electrode placement, stimulus dosing, repeated treatment schedules, age, vascular comorbidity, medication exposure, or patient-reported cognitive outcomes. Glial activation is also biologically ambiguous. It may reflect adaptive repair, metabolic support, synaptic remodeling, inflammatory activation, or nonspecific seizure-related stress. For this reason, glial mechanisms should be treated as plausible translator pathways requiring human validation, not as established mediators of antidepressant response.

The postictal window must become durable to produce remission. Synaptic, trophic, structural, and epigenetic mechanisms are therefore positioned as downstream consolidation processes (59, 65). BDNF, VEGF, hippocampal plasticity, stress-related methylation signals, and microRNAs may contribute to whether repeated postictal windows consolidate into recovery (59, 65, 67, 97). This ordering preserves neuroplasticity as a central element of the model, but places it where it is most clinically plausible: as a mechanism of durability, relapse vulnerability, and memory-network risk rather than as a complete explanation for immediate postictal reorientation (63, 68).

## 5 Clinical observations as explanatory constraints for the model

The following clinical observations should be understood as explanatory constraints rather than as direct proof of the postictal systems window hypothesis. Rapid improvement, response in psychotic or melancholic depression, treatment resistance, relapse vulnerability, and cognitive adverse effects are clinical phenomena that any adequate model of ECT should explain. The proposed framework adds value only if it explains these observations with greater temporal and mechanistic precision than simpler single-domain accounts, and if it generates testable predictions that can be compared with those accounts.

The postictal systems window hypothesis accounts for rapid improvement by placing early change in network interruption, hemodynamic-metabolic transition, and acute biological recoupling rather than in slow structural change alone (13, 74). A patient may become less suicidal, less psychotically preoccupied, less psychomotorically inhibited, or less agitated before hippocampal volume change or epigenetic consolidation could plausibly account for the full effect (25, 26, 98). Early response may reflect loosening of rigid depressive states during a biologically permissive postictal period. In this framework, early postictal coordination is the clinically relevant candidate event, whereas later plasticity and structural change are interpreted as consolidation processes rather than immediate explanations for early clinical improvement (13, 74).

The same model explains delayed remission across a course. Repeated windows may progressively weaken depressive allostasis and permit consolidation through synaptic, trophic, endocrine, immune-metabolic, structural, and epigenetic mechanisms (13). Each treatment is not merely another seizure. It is another opportunity for a pathological state to be interrupted and reorganized under altered neural, neurovascular, and systemic conditions.

High efficacy in psychotic and melancholic depression is also coherent with the model. These syndromes frequently involve psychomotor disturbance, neurovegetative collapse, insomnia, appetite disturbance, endocrine dysregulation, delusional meaning, and severe cognitive-affective rigidity (99–101). A treatment that simultaneously interrupts network activity, challenges HPA-axis dynamics, engages immune-metabolic signaling, and permits plastic consolidation is better matched to such syndromes than a single-transmitter intervention (100, 102).

Treatment-resistant depression may respond because pharmacological resistance is not the same as resistance to state reorganization (103, 104). Sequential medications may fail when a depressive episode is sustained by a cross-level loop involving brain networks, sleep, inflammation, endocrine tone, metabolic state, and behavior (4, 105). ECT may disrupt this loop directly by repeatedly imposing a coordinated postictal transition.

The model clarifies cognitive adverse effects by linking them to specific dimensions of the postictal window rather than treating them as an external complication. Antidepressant efficacy is hypothesized to depend primarily on a window with sufficient but contained amplitude and high coordination: postictal network interruption and recovery, hemodynamic-metabolic support, reorientation, and endocrine or immune-metabolic coupling should align closely enough to loosen rigid depressive self-reference, salience, psychomotor, and neurovegetative loops (13, 74, 83). This therapeutic effect should not require maximal seizure intensity, maximal stimulus dose, or maximal hippocampal exposure.

Cognitive adverse effects, by contrast, are hypothesized to depend more strongly on duration and anatomical distribution. ECT-related cognitive outcomes are heterogeneous: acute disorientation and impaired new learning commonly occur around the treatment course, many objective measures recover after treatment, but retrograde autobiographical memory loss and patient-reported cognitive burden may be more persistent and clinically meaningful (41, 42). In the present model, prolonged reorientation, persistent postictal slowing, delirious recovery, or disproportionate exposure of medial temporal, hippocampal, and autobiographical memory networks would indicate a window that is excessive, prolonged, or anatomically unfavorable (15, 41, 68, 106, 107).

In this sense, electric-field distribution and hippocampal involvement are not simply incorporated into the framework after the fact. They operationalize the anatomical-distribution dimension of the postictal systems window and specify where postictal recovery and plasticity processes are occurring. This distinction allows a more precise prediction than a general efficacy-toxicity trade-off. Antidepressant response should be partly dissociable from cognitive burden when technique preserves adequate postictal coordination while reducing excessive duration or medial temporal exposure. Conversely, cognitive burden should increase when the window is prolonged, poorly contained, or weighted toward autobiographical memory networks, even if depressive symptoms improve. ECT-related cognitive outcomes are therefore not only safety endpoints; they are a central falsification domain for the model (1, 41–45, 106, 107).

Finally, the model accounts for variability by electrode placement, stimulus dose, pulse width, seizure quality, anesthetic agent, age, inflammatory state, vascular disease, and medical comorbidity. Each can alter window amplitude, coordination, distribution, or duration (1, 108, 109). Bilateral ECT may produce a broader and more robust window, sometimes increasing efficacy and cognitive risk (1). Right unilateral ultrabrief ECT may narrow exposure and reduce memory burden, but may require careful dosing and monitoring to preserve efficacy (1, 110). Anesthetic agents may alter seizure expression, hemodynamics, endocrine release, and recovery (108, 109, 111). Age and vascular disease may increase both responsiveness and vulnerability (3, 99).

## 6 Testable predictions and falsification

The hypothesis is useful only if it can fail. **Table 4** lists falsifiable predictions. The central prediction is that early postictal dynamics, especially after the first one to three treatments, will predict later clinical response and cognitive outcome better than isolated baseline variables or seizure duration alone (14, 84, 112).

**Table 4:** Falsifiable predictions and operational tests of the postictal systems window hypothesis.

Prediction	Operational test/data stream	Model-consistent finding	Finding that would weaken the claim	Implication for the hierarchy
Early postictal dynamics predict later remission	Model EEG recovery, time to orientation, early symptom change and timed blood markers after ECT sessions 1-3; adjust for baseline severity, phenotype, technique, anesthesia, medications and seizure duration.	Coordinated early postictal curves predict remission, time to response and suicidality reduction beyond conventional clinical and procedural predictors.	No reproducible association between early postictal trajectories and later clinical outcome after adequate adjustment.	Supports the postictal window as the clinically informative event rather than seizure occurrence alone.
A prespecified composite outperforms isolated markers	Compare a postictal-window index capturing amplitude, coordination, distribution and duration against seizure duration, PSI, cortisol/prolactin, CRP/IL-6, BDNF or hippocampal volume alone.	The composite improves discrimination, calibration and clinical utility beyond single biomarkers and standard ECT variables.	One isolated marker consistently outperforms a well-built, internally validated multimodal index across independent cohorts.	Tests whether the model is genuinely systems-level rather than a relabeled single-biomarker hypothesis.
Responders show coordinated acute biology	Estimate cross-domain coupling among EEG suppression and recovery, hemodynamic-metabolic curves, endocrine response, immune-metabolic markers and reorientation.	Responders show a brisk, contained and temporally coupled postictal response; nonresponders show weak, uncoupled, delayed or prematurely resolving dynamics.	Responders and nonresponders have indistinguishable early postictal curves, or only nonspecific amplitude differs.	Places coordination and recovery quality near the core of the mechanism.
Cognitive burden reflects excessive, prolonged or mistargeted exposure	Relate time to reorientation, persistent postictal slowing, autobiographical memory, medial temporal electric-field exposure and hippocampal/network imaging to cognition.	Memory burden correlates with delayed recovery, prolonged slowing or medial temporal exposure, and can be partly dissociated from antidepressant efficacy.	Cognitive effects are unrelated to recovery, anatomical exposure or window duration, or remain inseparable from efficacy despite optimized technique.	Separates therapeutic engagement from toxicity and makes cognition a central falsification domain.
Technique and anesthesia shape window quality	Model electrode placement, pulse width, stimulus dose relative to threshold, treatment frequency, anesthetic agent and concomitant medications against window-index components.	Procedural variables alter amplitude, coordination, distribution or duration, and these changes mediate differences in response and cognitive outcome.	Procedural variables do not alter measurable window biology, or seizure duration alone fully explains technique-outcome relations.	Reframes optimization as shaping the postictal window, not simply maximizing seizure length or stimulus intensity.
Neurovascular dynamics are core; BBB/NVU permeability remains secondary	Use ASL, diffuse optical or NIRS measures of perfusion/oxygenation; test DCE-MRI, S100B, GFAP, NfL, albumin quotient or CSF only in justified substudies.	Postictal perfusion/oxygenation recovery relates to EEG recovery, reorientation and outcome. BBB/NVU signals, if present, are selective, brief and not universal.	No structured neurovascular-hemodynamic dynamics are detectable and none relate to recovery or outcome. Negative BBB/NVU findings alone do not falsify the core model.	Protects the model from overclaiming BBB opening while preserving a testable brain-body interface.
Repeated windows consolidate response and maintenance stabilizes vulnerability	Track symptom, cognitive and biomarker trajectories across the acute course, follow-up and maintenance ECT; sample before maintenance sessions where feasible.	Early windows accumulate into durable remission; biological or clinical drift precedes relapse; maintenance ECT reinduces a smaller stabilizing window.	Relapse risk and maintenance benefit are unrelated to early or longitudinal biological trajectories.	Links acute postictal events to consolidation, relapse vulnerability and continuation-care biology.

Note. Primary analyses should be prespecified and should adjust, at minimum, for age, baseline depression severity, psychotic/melancholic/catatonic or retarded features, electrode placement, pulse width, stimulus dose relative to seizure threshold, anesthetic agent, medication exposure and relevant medical comorbidity. Negative BBB/NVU results should weaken only the exploratory interface component unless core EEG, clinical reorientation and neurovascular-hemodynamic dynamics are also absent. Abbreviations: ASL, arterial spin labeling; BBB, blood-brain barrier; BDNF, brain-derived neurotrophic factor; CRP, C-reactive protein; CSF, cerebrospinal fluid; DCE-MRI, dynamic contrast-enhanced magnetic resonance imaging; ECT, electroconvulsive therapy; EEG, electroencephalography; GFAP, glial fibrillary acidic protein; IL-6, interleukin-6; NfL, neurofilament light chain; NIRS, near-infrared spectroscopy; NVU, neurovascular unit; PSI, postictal suppression index.

First, early postictal biomarkers should predict later remission. The most informative measures are likely to be curves rather than single values: postictal suppression and recovery, orientation recovery, cortisol or prolactin trajectories, inflammatory and kynurenine shifts, lactate, and early psychomotor or suicidality change (14, 84, 85). A consistent absence of association between early postictal dynamics and later outcome would weaken the model.

Second, a prespecified composite postictal-window index should outperform single biomarkers. Postictal suppression alone, cortisol alone, CRP alone, BDNF alone, or hippocampal volume alone should be less predictive than an index capturing amplitude, coordination, distribution, and duration (31). A serious challenge would be a reproducible finding that one isolated marker predicts response and cognitive outcome better than a well-built multimodal index.

Third, responders should show a coordinated acute biological response after early treatments: adequate seizure expression, meaningful but contained postictal suppression, interpretable endocrine activation, time-limited inflammatory-metabolic change, and recovery without prolonged delirium (14, 69). Nonresponders may show a small, uncoupled, or prematurely resolving window. Patients with cognitive toxicity may show an excessive, prolonged, or hippocampal-weighted window (107).

Fourth, cognitive adverse effects should correlate with excessive or prolonged window exposure rather than antidepressant efficacy itself. Relevant measures include orientation time, autobiographical memory, hippocampal or medial temporal electric-field exposure, neurovascular changes, and memory-network involvement (107, 113). Antidepressant response should not require maximal hippocampal exposure. If cognitive effects were inseparable from antidepressant efficacy across optimized techniques, the model would be weakened (107, 114).

Fifth, technique optimization should shape window quality, not merely lengthen the seizure. Electrode placement, pulse width, stimulus dose, treatment frequency, anesthetic choice, and medication management should be studied as methods of shaping amplitude, coordination, distribution, and duration (1, 115). A model-consistent trial would compare symptoms and cognition together with early postictal-window indices across techniques (18, 45, 115).

The strongest test of the hypothesis is not whether individual domains change after ECT, but whether their early coordination predicts outcome better than simpler competing models.

## 7 Proposed biomarker framework

### 7.1 A feasible core panel

A useful biomarker framework must be clinically implementable. A maximal mechanistic battery is not the same as a practical study. We therefore propose a two-tier framework. The core tier should be feasible in a busy ECT service: clinical ratings, time to orientation, routine ECT EEG metrics, and a small number of timed blood markers (31, 100). The exploratory tier should be reserved for mechanistic cohorts: advanced imaging, broader omics, cerebrospinal fluid where clinically justified, and electric-field modeling (107, 113). **Table 5** summarizes candidate markers, timing, interpretation, and confounders.

**Table 5:** Candidate measurement framework for the postictal systems window hypothesis: core panel and exploratory mechanistic substudies.

Tier	Domain	Operational package and timing	Interpretive use and key design controls
Core	ECT procedure and EEG	Record at every treatment: electrode placement, pulse width, stimulus dose relative to threshold, anesthetic regimen, seizure threshold, ictal morphology and amplitude, motor and EEG seizure duration, postictal suppression index, postictal slowing and EEG recovery slope. Detailed extraction: sessions 1, 3, 6, and final acute treatment.	Interpretive use: minimum physiological readout of seizure induction, window amplitude, duration, and containment. Design controls: anesthetic agent and dose, benzodiazepines, anticonvulsants, lithium, age, electrode placement, stimulus dose, threshold drift and device algorithms. Do not use seizure duration as the sole adequacy marker.
Core	Clinical and cognitive recovery	Assess at baseline, after early sessions, end of course, 2–4 weeks, and 3 months; orientation after every treatment. Include time to reorientation, acute confusion or delirium, MADRS or HDRS, structured suicidality, psychomotor/catatonia ratings where indicated, psychosis items, sleep, appetite, global functioning, brief cognition, autobiographical memory and patient-reported cognition.	Interpretive use: links the biological window to patient-visible benefit, harm and relapse vulnerability. Design controls: baseline cognitive impairment, depression severity, psychosis, delirium, sleep deprivation, medical illness, medication changes, rater drift and practice effects; use fixed instruments and consistent timing.
Core	Endocrine dynamics	Timed blood sampling: pre-treatment and approximately 30 min, 2 h and 24 h after sessions 1 and 3; optional session 6. Cortisol or prolactin as the pragmatic core; ACTH and DHEA/DHEA-S only if feasible.	Interpretive use: HPA-axis pulsatility, systemic engagement and recovery flexibility. Design controls: circadian timing, fasting/sleep state, corticosteroid exposure, endocrine disease, medications, anesthetic agent and sample handling.
Core	Inflammatory state	Sampling: baseline, early postictal time points, session 6, end of course and follow-up. CRP or IL-6 as feasible core markers; optional TNF-alpha, IL-8 and IL-10 in larger cohorts.	Interpretive use: inflammatory phenotype and dynamic immune engagement, not a direct central immune readout. Design controls: infection, obesity, smoking, autoimmune disease, vascular disease, NSAIDs, statins and assay platform.
Extended core	Metabolic-kynurenine	Sampling: baseline and timed samples after sessions 1 and 3, with selected later samples. Tryptophan, kynurenine, kynurenic acid, 3-hydroxykynurenine and lactate.	Interpretive use: immune-metabolic recoding and glutamatergic/oxidative-stress links. Design controls: diet, fasting status, renal and hepatic function, exercise, acute illness, antidepressants and other medications.
Exploratory	Neuroimaging and hemodynamics	Substudy timing: baseline, selected early post-treatment window, end of course and follow-up. ASL-MRI, diffuse optical/NIRS monitoring, DCE-MRI, resting-state fMRI, DTI/DTI-ALPS and electric-field modelling.	Interpretive use: perfusion, oxygenation, network reorganization, anatomical exposure and exploratory BBB/NVU dynamics. Design controls: motion, sedation, vascular disease, scanner/site effects, contrast eligibility and acquisition harmonization.
Exploratory	BBB/NVU and injury-interface markers	Sampling: baseline; 30 min, 2 h and 24 h after sessions 1 and 3; end of course. S100B, GFAP and NfL; albumin quotient only where CSF is clinically available.	Interpretive use: astrocytic stress, neuronal-injury signal and barrier/interface dynamics; not validated clinical tests. Design controls: age, trauma, neurodegeneration, systemic illness, extracerebral sources, renal function and assay platform.
Exploratory	Plasticity and epigenetics	Sampling: baseline, session 6, end of course and follow-up, with selected early samples. BDNF, VEGF, FKBP5, NR3C1, TNKS-related methylation and selected microRNAs.	Interpretive use: consolidation of repeated windows and stress-plasticity regulation. Design controls: blood-cell composition, antidepressant exposure, age, tissue specificity, batch effects and sample handling; avoid inferring rapid causality from late molecular change.

Note. The core panel is intended for pragmatic prospective ECT cohorts and for construction of a prespecified postictal-window index. Extended core and exploratory measures should be used in adequately powered mechanistic substudies and should not be presented as validated clinical tests. BBB/NVU measures are exploratory interfaces, not required mediators of the hypothesis. Abbreviations. ACTH, adrenocorticotropic hormone; ASL, arterial spin labeling; BBB, blood-brain barrier; BDNF, brain-derived neurotrophic factor; CRP, C-reactive protein; CSF, cerebrospinal fluid; DCE-MRI, dynamic contrast-enhanced magnetic resonance imaging; DHEA, dehydroepiandrosterone; DTI, diffusion tensor imaging; DTI-ALPS, diffusion tensor imaging along the perivascular space; EEG, electroencephalography; ECT, electroconvulsive therapy; fMRI, functional MRI; GFAP, glial fibrillary acidic protein; HDRS, Hamilton Depression Rating Scale; HPA, hypothalamic-pituitary-adrenal; IL, interleukin; MADRS, Montgomery-Asberg Depression Rating Scale; NfL, neurofilament light chain; NIRS, near-infrared spectroscopy; NVU, neurovascular unit; PSI, postictal suppression index; VEGF, vascular endothelial growth factor.

The core panel should include seizure quality, seizure duration, postictal suppression index, EEG recovery slope, time to reorientation, depression severity, suicidality, psychomotor status, psychosis where relevant, sleep and appetite items, cortisol or prolactin response, CRP or IL-6, and selected kynurenine-pathway metabolites where laboratory capacity permits (69, 84, 85). This panel is not intended to diagnose response after one treatment. It is intended to capture whether the patient is entering a coordinated postictal state.

### 7.2 Exploratory mechanistic substudies

The exploratory panel should test vulnerable or biologically specific components of the model. Arterial spin labeling MRI can examine perfusion (116, 117). Diffuse optical monitoring can track hemodynamic and oxygenation changes around treatment where available. Dynamic contrast-enhanced MRI can examine BBB/NVU permeability where ethically and technically appropriate. Resting-state functional MRI can assess network reorganization (118). Diffusion imaging, DTI-ALPS, and electric-field modeling can examine anatomical exposure, white matter, and perivascular diffusion hypotheses (107, 113, 119). GFAP, neurofilament light chain, S100B, albumin quotient, broad cytokine panels, BDNF, VEGF, methylation profiles, and microRNAs should be interpreted cautiously and not treated as validated clinical tests (59, 120).

### 7.3 Constructing the postictal-window index

The postictal-window index should be specified *a priori* before model fitting, including candidate variables, sampling windows, transformations, and weighting strategy. A pragmatic structure is to define four separable domain scores: amplitude, coordination, distribution, and duration. These terms should be treated as operational domains rather than descriptive metaphors.

The rationale for this four-domain structure is both biological and pragmatic. Biologically, the postictal window is conceptualized as a transient perturbation that can be described by its magnitude across relevant systems, the temporal coupling among those systems, the anatomical and procedural exposure through which the perturbation is expressed, and the time course over which the system recovers or remains altered. Pragmatically, these four domains organize variables that can be measured in future ECT cohorts and tested against simpler predictors such as seizure duration, postictal suppression alone, baseline severity, clinical phenotype, electrode placement, stimulus dose, anesthetic agent, and medication exposure. The domains are not assumed to be fully independent. Overlap and interaction are expected: amplitude may matter differently depending on duration, anatomical distribution may determine whether a coordinated window is therapeutic or cognitively costly, and coordination itself depends on the temporal relationship among electrophysiological, clinical, hemodynamic-metabolic, endocrine, and immune-metabolic curves. The purpose of the index is therefore not to impose artificial biological separations, but to make the hypothesis measurable, reproducible, and testable against competing explanations.

- Amplitude refers to the magnitude of the acute postictal perturbation. In a core clinical panel, it may include seizure expression, seizure-quality measures, postictal suppression magnitude, early postictal slowing, and peak endocrine response. Where available, hemodynamic or perfusion response may provide an additional amplitude measure (14, 117).
- Coordination refers to temporal coupling across domains. It may include the alignment of EEG recovery, time to orientation, endocrine response, inflammatory-metabolic change, and early clinical trajectory. A coordinated window would not require every marker to change in the same direction, but would require interpretable temporal coupling among electrophysiological, clinical, and systemic recovery curves (69, 84, 85).
- Distribution refers to anatomical and procedural exposure of the postictal window. In routine cohorts, this may be approximated by electrode placement, pulse width, stimulus dose relative to seizure threshold, seizure threshold, and treatment technique. In mechanistic substudies, it may be refined using electric-field estimates, medial temporal or hippocampal exposure, perfusion distribution, and network-level imaging measures (107, 114, 121).
- Duration refers to the persistence of the postictal state before recovery or transition to a new equilibrium. Core measures may include EEG recovery slope, time to reorientation, duration of acute confusion, persistence of postictal slowing, and delayed clinical recovery. Extended protocols may also examine the time to normalization of endocrine, inflammatory-metabolic, lactate, perfusion, or oxygenation measures (15, 84).

**Table 6** operationalizes these four domains and distinguishes core clinically feasible measures from extended mechanistic measures.

**Table 6:** Operationalizing the four dimensions of the postictal-window index.

Dimension.	Operational definition	Core clinically feasible measures	Extended mechanistic measures
Amplitude	Magnitude of the acute postictal perturbation after an adequate ECT-induced seizure.	EEG seizure quality; EEG seizure duration; postictal suppression magnitude; early postictal slowing; peak change in cortisol or prolactin; early change in orientation or alertness.	ASL-MRI or NIRS perfusion/oxygenation peak; lactate peak; broader endocrine panel; acute inflammatory or kynurenine-pathway change.
Coordination	Temporal coupling among electrophysiological, clinical, hemodynamic-metabolic, endocrine, and immune-metabolic responses.	Alignment between postictal suppression, EEG recovery slope, time to reorientation, and early clinical change; concordant direction of early symptom, cognition, and recovery measures.	Cross-domain coupling of EEG, NIRS/ASL, cortisol/prolactin, CRP/IL-6, kynurenine metabolites, lactate, and early network or perfusion change.
Distribution	Anatomical and procedural exposure of the postictal window across brain regions and networks.	Electrode placement; pulse width; stimulus dose relative to seizure threshold; seizure threshold; unilateral versus bilateral technique; treatment frequency.	Electric-field modeling; medial temporal or hippocampal exposure; resting-state network changes; perfusion distribution; individualized anatomical modeling.
Duration	Persistence of postictal electrophysiological, clinical, hemodynamic, and systemic deviations before recovery or consolidation.	Time to reorientation; duration of postictal confusion; EEG recovery slope; persistence of postictal slowing; recovery-room clinical trajectory.	Time to normalization of perfusion/oxygenation, lactate, cortisol/prolactin, inflammatory or kynurenine markers; longer inter-session recovery and cognitive trajectory.

Note. The index should initially be developed from a minimal core variable set available in routine ECT practice. Extended imaging, omics, BBB/NVU, glial, and advanced hemodynamic measures should be tested only for incremental value in adequately powered mechanistic substudies. All variables, time points, transformations, and weighting procedures should be prespecified before model fitting. Abbreviations: ASL-MRI, arterial spin labeling magnetic resonance imaging; CRP, C-reactive protein; ECT, electroconvulsive therapy; EEG, electroencephalography; IL-6, interleukin-6; MRI, magnetic resonance imaging; NIRS, near-infrared spectroscopy.

Each domain should first be reported separately before being combined into a total postictal-window index. The primary analysis should then compare the composite against isolated markers and standard clinical-procedural predictors, including seizure duration, postictal suppression alone, baseline severity, clinical phenotype, electrode placement, pulse width, stimulus dose, anesthetic agent, and medication exposure (31, 99). The index should be penalized for overfitting, internally validated, and ideally tested in an external cohort. Without prespecification and validation, the model would risk becoming a descriptive vocabulary rather than a reproducible predictive framework.

For reproducibility, each domain should be reported separately before being combined into a total postictal-window index. Variables should be standardized within study protocols, adjusted for baseline values where appropriate, and interpreted in relation to electrode placement, stimulus dose, pulse width, anesthetic agent, medication exposure, age, vascular burden, and baseline cognitive status. The initial index should use prespecified weights or penalized modeling with internal validation, and its incremental value should be tested against simpler predictors such as seizure duration, postictal suppression alone, baseline severity, clinical phenotype, and procedural variables. External validation in an independent ECT cohort is required before the index can be considered for clinical translation.

### 7.4 Feasibility, cost, and validation constraints

The proposed biomarker framework should not be interpreted as a routine clinical package ready for immediate implementation in all ECT services. Its purpose is to define a staged research strategy. The pragmatic core panel should remain deliberately narrow: ECT procedural variables, routine EEG-derived seizure and postictal measures, time to reorientation, standardized symptom and cognitive ratings, and a limited number of timed blood markers. These measures are more likely to be feasible in ordinary clinical services and are less likely to disrupt care. By contrast, ASL-MRI, diffuse optical or NIRS monitoring, DCE-MRI, resting-state fMRI, omics, CSF markers, and electric-field modeling should be reserved for adequately resourced mechanistic substudies.

Cost and workflow constraints are substantial. Timed postictal sampling requires staff availability immediately before and after ECT, coordination with anesthesia and recovery-room procedures, sample processing capacity, and reliable documentation of exact sampling times. Repeated blood draws may be difficult in older, medically frail, catatonic, agitated, severely suicidal, or cognitively impaired patients. Advanced imaging adds scanner cost, transport burden, motion artefact, contrast eligibility issues, and the practical difficulty of studying acutely ill patients close to treatment sessions. These constraints make it unrealistic to expect a full multimodal battery in routine practice.

Inter-center variability is another major barrier. ECT devices, EEG acquisition, proprietary seizure-quality algorithms, electrode placement, pulse width, stimulus dosing strategy, treatment frequency, anesthetic agent, benzodiazepine or anticonvulsant exposure, lithium use, recovery-room procedures, and cognitive monitoring differ substantially between services. Biomarker studies are also vulnerable to variation in circadian timing, fasting state, tube type, processing delay, storage temperature, assay platform, batch effects, and local laboratory procedures. Without harmonized protocols, apparent biological differences may reflect site effects rather than true postictal-window biology.

The statistical challenges are equally important. A postictal-window index would combine repeated measures, time-dependent curves, clinical variables, EEG features, and biomarkers that are often correlated and incomplete. This creates a high risk of overfitting, multiple-testing artefacts, and unstable variable weighting, especially in small single-center cohorts. The index should therefore be prespecified before model fitting, tested first with a minimal core variable set, and evaluated against simpler clinical and procedural predictors. Analyses should account for repeated measures, missing data, center effects, anesthesia, electrode placement, stimulus dose, medication exposure, baseline severity, age, and medical comorbidity.

A credible validation pathway would require stepwise development. First, a parsimonious core index should be derived in a prospective cohort using clinically obtainable measures. Second, its discrimination, calibration, and incremental value over standard predictors should be tested with internal validation, preferably using bootstrapping or cross-validation with penalization. Third, the model should be externally validated in an independent ECT cohort before any clinical claims are made. Exploratory imaging, omics, BBB/NVU, or glial markers should be tested for incremental value only after the core index has shown reproducible performance. Until such validation is available, the postictal-window index should be regarded as a research tool rather than a clinical decision instrument.

## 8 Proposed study design to test the hypothesis

A feasible prospective translational study would enroll adults with major depressive disorder referred for an acute ECT course as part of standard care (31, 122). The cohort should include severe, melancholic, psychotic, suicidal, catatonic or retarded, and treatment-resistant presentations (1). Inclusion should require a depressive episode of sufficient severity to justify ECT. Exclusion criteria should be limited to conditions that make interpretation unsafe or impossible: acute neurological injury, active systemic infection, major immunosuppressive or corticosteroid exposure when biomarkers are central, and contraindications to optional MRI or contrast procedures.

The design should not distort clinical care. Electrode placement, pulse width, anesthetic agent, stimulus dosing, and treatment frequency should be selected by the treating team and recorded precisely (122). A two-tier sample would be realistic: 120–180 patients for clinical, EEG, cognitive, and blood-based modeling, with an imaging or hemodynamic substudy of 40–60 patients. Older and medically complex patients should not be excluded unless safety requires it, because they are often the patients in whom ECT is clinically decisive (31, 123).

Sampling should be centered on the early postictal period. Before session 1, patients would undergo baseline ratings, cognitive testing, medication and comorbidity review, and standardized blood sampling (122, 124). After sessions 1 and 3, blood would be drawn immediately before ECT and at approximately 30 minutes, 2 hours, and 24 hours after treatment (125). Session 6 would include pre-treatment and early post-treatment sampling. End-of-course and follow-up assessments at 2–4 weeks and 3 months would capture remission, relapse, and cognitive recovery (41). The exact time points can be adapted to ward logistics, but the biological principle is non-negotiable: early postictal curves are required.

Clinical measures should include the Montgomery-Asberg Depression Rating Scale or Hamilton Depression Rating Scale, a structured suicidality measure, psychosis ratings where relevant, catatonia or psychomotor-retardation ratings when indicated, sleep and appetite items, and global functioning (122, 126). Cognitive assessment should include time to orientation after each treatment, a brief global screen, verbal learning and delayed recall, processing speed, executive function, and an autobiographical memory measure (41, 127). Patient-reported cognitive experience should be collected rather than inferred from objective tests alone (127).

EEG measures should include seizure duration, seizure threshold when available, ictal amplitude and morphology, postictal suppression index, postictal slowing, and recovery slope (84). Blood biomarkers should follow the two-tier principle: a core set of cortisol or prolactin, CRP or IL-6, and selected kynurenine-pathway metabolites where feasible; expanded panels including ACTH, DHEA/DHEA-S, TNF-alpha, IL-8, IL-10, lactate, BDNF, VEGF, S100B, GFAP, neurofilament light chain, methylation, and microRNAs only in adequately powered substudies (59, 69, 122, 128).

The primary clinical endpoint should be remission at the end of the acute ECT course, defined prospectively by a standard depression scale threshold. A co-primary mechanistic endpoint should test whether a prespecified postictal-window index after sessions 1–3 predicts remission beyond baseline severity, age, psychotic features, electrode placement, pulse width, stimulus dose, anesthetic agent, medications, and medical comorbidity (31, 126, 129). Secondary endpoints should include time to response, reduction in suicidal ideation, psychomotor improvement, psychosis resolution, orientation recovery, objective cognition, autobiographical memory, patient-reported cognition, and relapse during follow-up (41, 130).

The analytic strategy should model trajectories rather than isolated pre-post differences (129, 130). Mixed-effects models, latent class or trajectory methods, and regularized prediction can be used, but the composite index must be prespecified and internally validated (130, 131). Biomarker features should include peak, slope, area under the curve, recovery time, and cross-domain coupling (124). The model is supported if early coordinated postictal dynamics predict clinical response and cognitive outcome beyond conventional procedural and clinical variables.

## 9 Clinical implications

If supported, the postictal systems window model would not immediately prescribe a new ECT technique. Its first value would be conceptual. It would shift clinical thinking from seizure occurrence to postictal recovery quality. It would also make adverse cognitive effects mechanistically central rather than an inconvenient complication placed outside the efficacy model (1, 132).

Patient selection could become more biologically informed. Severe melancholic, psychotic, suicidal, retarded, catatonic, or inflammatory-metabolic depressive phenotypes may be especially relevant because they represent system-level dysregulation (31). This does not mean ECT should be restricted to those phenotypes. It means future studies should ask whether different depressive states enter different postictal windows.

Response prediction could move from baseline-only variables toward early dynamic assessment. After the first few treatments, clinicians might eventually have evidence that a patient is showing a coordinated therapeutic window, a weak window suggesting nonresponse, or an excessive window suggesting cognitive vulnerability (133, 134). Such tools are not yet available for routine use, but the model indicates what should be measured.

Technique personalization would be reframed. The goal would be not the longest seizure or the greatest stimulus intensity, but a window with the right amplitude, coordination, distribution, and duration for the individual patient (1, 3). Electrode placement, pulse width, dosing strategy, treatment frequency, anesthetic agent, and concomitant medications would be studied as variables that shape the postictal window (3, 106, 135).

Magnetic seizure therapy may provide an informative translational test case for this framework. MST induces a therapeutic seizure through a different field geometry than conventional ECT and has been developed partly to reduce cognitive adverse effects while preserving antidepressant efficacy. Recent randomized evidence comparing MST with right unilateral ultrabrief ECT is therefore directly relevant to the model (45). Within the postictal systems window framework, MST would be expected to preserve sufficient postictal amplitude and coordination for antidepressant response while altering anatomical distribution and possibly duration in a way that reduces medial temporal or autobiographical memory-network exposure. MST therefore provides a practical opportunity to test whether efficacy and cognitive burden depend on partly separable properties of the same postictal window rather than on seizure induction alone. If MST preserves antidepressant benefit while reducing delayed reorientation, autobiographical memory burden, or medial temporal exposure, this would support the model’s distinction between therapeutic coordination and cognitive-risk distribution. Conversely, if MST outcomes are unrelated to postictal recovery, distribution, or duration, the framework would require revision.

Cognitive mitigation would become part of mechanism-based care. Prolonged reorientation, early autobiographical memory complaints, excessive postictal confusion, or evidence of disproportionate medial temporal exposure could prompt reassessment of technique, spacing, dose, anesthetic choice, and medications (1, 106). From a clinical perspective, time to reorientation, patient-reported memory difficulties, and structured autobiographical-memory assessment may be among the most useful bedside signals that the postictal window is becoming excessive, prolonged, or anatomically unfavorable. This framing respects patient experience. Memory effects are not peripheral to ECT research; they are part of the therapeutic-window problem (132).

Continuation and maintenance ECT can also be reinterpreted. Relapse after acute ECT may reflect drift back toward depressive allostasis after the new equilibrium remains unstable (1, 37). Maintenance treatment may work by periodically reinducing a smaller stabilizing window (136, 137). This is a hypothesis, not a recommendation. It suggests that maintenance schedules should eventually be studied with the same dynamic biomarkers used in acute treatment.

Combination strategies may be tested more rationally. Anti-inflammatory, chronobiological, sleep-focused, psychopharmacological, cognitive, or neuroprotective interventions might be timed to the postictal or consolidation phase (69, 70). Such combinations should not be adopted without trials. The model supplies a rationale for timing and measurement, not a license for speculative augmentation.

## 10 Limitations and alternative explanations

The proposed model is intentionally integrative, and that creates risk. A model that includes networks, hemodynamics, endocrine response, inflammation, metabolism, glia, synapses, epigenetics, and BBB/NVU biology can become unfalsifiable unless it specifies timing and hierarchy. The model would be uninteresting if it merely renamed known postictal biology. Its value depends on whether early window quality predicts response and cognitive burden better than simpler explanations.

The main risk of the proposed model is conceptual over-integration. Several simpler explanations remain plausible. A seizure-quality model would argue that seizure morphology, seizure duration, and postictal EEG suppression are sufficient to explain response. A network-first model would interpret endocrine, inflammatory, metabolic, and vascular changes as downstream correlates of circuit reorganization. A neuroplasticity-consolidation model would place the main mechanism in repeated synaptic, trophic, structural, and epigenetic change rather than in the early postictal period. A stress-response model would treat endocrine and inflammatory activation as nonspecific systemic physiology. An inflammatory-metabolic subgroup model would argue that baseline biological phenotype, rather than postictal coordination, explains differential response. Finally, a procedural-confounding model would attribute apparent window effects to electrode placement, pulse width, stimulus dose, anesthetic agent, medications, age, vascular burden, or baseline cognition.

The postictal systems window hypothesis must therefore be tested against these alternatives rather than assumed to incorporate them. It would be weakened if seizure-quality variables, network measures, baseline inflammatory-metabolic phenotype, endocrine response, neuroplasticity markers, or procedural variables predicted remission and cognitive burden as well as or better than a prespecified multimodal postictal-window index. Its added value depends on demonstrating that early coordinated postictal dynamics provide incremental explanatory or predictive information beyond these simpler accounts.

A balanced interpretation also requires explicit attention to negative, null, and contradictory findings. Postictal suppression and EEG recovery are clinically attractive because they are close to the induced seizure and can be measured during routine ECT, but their associations with antidepressant outcome and cognitive effects have not been uniform. Postictal suppression should therefore be treated as one accessible signal of the postictal state, not as a validated surrogate endpoint or a sufficient response biomarker. A model in which postictal suppression alone explains remission would be simpler than the present hypothesis; the postictal systems window is justified only if multimodal recovery dynamics add predictive value beyond isolated EEG measures (14, 16, 18, 83–85).

The same caution applies to endocrine markers. ECT can acutely alter cortisol, ACTH, prolactin, and related hormones, but these responses are not specific to antidepressant efficacy. They are influenced by seizure quality, anesthetic agent, circadian timing, baseline HPA-axis state, medical illness, medications, and the stress physiology of the procedure itself. A brisk hormonal response may indicate systemic engagement, but a single cortisol or prolactin value cannot establish mechanism. Negative or inconsistent endocrine-response studies would not by themselves falsify the model, but they would argue against treating HPA-axis activation as a central or sufficient mediator (56–58, 125).

Inflammatory and kynurenine-pathway findings are also heterogeneous. Although some studies and meta-analytic signals suggest that baseline CRP, IL-6, cytokines, or kynurenine-pathway metabolites may relate to ECT response in subgroups, peripheral inflammatory markers are nonspecific and may not reflect central immune states. Acute inflammatory changes after ECT may represent physiological engagement, repair signaling, medication effects, comorbidity, obesity, infection, smoking, vascular disease, or assay-related variation rather than direct therapeutic mediation. Therefore, inflammatory markers should be framed as possible stratification and coupling signals, not as validated clinical predictors (69, 70, 72, 138, 139).

Neuroimaging evidence should be interpreted with similar restraint. ECT-associated changes in functional connectivity, perfusion, hippocampal or amygdala volume, and electric-field exposure are important, but they are not uniformly linked to symptom remission, and structural change is not equivalent to antidepressant mechanism. Hippocampal volume increase, for example, may reflect plasticity, edema, vascular or glial processes, medication effects, or mixed biological remodeling; it may also relate differently to clinical improvement and memory burden. Imaging findings are therefore best viewed as candidate anatomical and physiological readouts of the window rather than proof that any single structural or network change mediates response (13, 61, 63, 64, 73–78, 116–118).

Also, negative evidence has different implications depending on the domain. Failure to demonstrate gross BBB opening during routine ECT would weaken the BBB/NVU interface component but would not falsify the core model, because BBB/NVU permeability is treated as exploratory rather than necessary. In contrast, repeated failure of early postictal EEG recovery, reorientation, hemodynamic-metabolic dynamics, and early clinical change to predict remission or cognitive burden beyond conventional variables would directly challenge the postictal systems window hypothesis. The decisive test is therefore not whether individual markers change after ECT, but whether their early coordination provides incremental predictive value over simpler competing explanations (80, 81).

Psychotic melancholia, agitated suicidal depression, late-life vascular depression, inflammatory anhedonic depression, bipolar depression, and chronic nonmelancholic treatment-resistant depression may not share the same window biology (9, 31). ECT practice is also heterogeneous across electrode placement, pulse width, stimulus dosing, frequency, anesthesia, medications, and local technique (108). Studies that ignore these variables will not adequately test the hypothesis.

CRP, IL-6, TNF-alpha, IL-8, IL-10, kynurenine metabolites, BDNF, cortisol, S100B, GFAP, and neurofilament light chain are influenced by age, sex, body mass index, sleep, smoking, diet, infection, vascular disease, neurodegeneration, medications, circadian timing, sample handling, and assay platform (69, 122, 138). They may not reflect central processes (70). A biomarker that predicts response may still be a correlate of adequate physiological engagement rather than a causal mediator (69, 139).

BBB and NVU dynamics are especially difficult to measure in humans. Serum markers are nonspecific (81, 140). Cerebrospinal fluid sampling is rarely feasible. Dynamic contrast-enhanced MRI is logistically demanding and may be inappropriate for some patients. Current human evidence does not establish therapeutic BBB opening during ECT (80, 81). This component should therefore remain secondary and testable, not central to the claim.

Anesthesia is not a nuisance variable. It alters seizure threshold, cerebral physiology, autonomic response, endocrine release, and recovery (109). Age, medications, vascular comorbidity, seizure duration, seizure threshold, electrode placement, stimulus dose, and number of treatments can all influence biomarkers and outcomes (31, 141). These variables must be measured precisely.

Causality remains uncertain. Even if early postictal dynamics predict response, they may be markers of adequate brain engagement rather than mediators of antidepressant action (123). Conversely, early clinical improvement may alter sleep, appetite, inflammation, and endocrine tone, thereby changing biomarkers (70, 72). Experimental manipulation of the window will be difficult because ECT parameters cannot be varied solely for mechanistic curiosity when patients are severely ill. The hypothesis should proceed through prospective prediction, careful technique comparisons, and eventually mechanism-informed trials (122, 142).

The same translational caution applies to glial mechanisms. Animal electroconvulsive stimulation studies can identify astrocytic, microglial, vascular-endfoot, purinergic, and synaptic changes with a level of cellular resolution that is not feasible in routine human ECT research. However, these findings may be influenced by species-specific neuroimmune biology, stimulation paradigms, seizure burden, anesthesia, tissue-sampling time, and experimental stress. They should not be interpreted as direct evidence that glial activation mediates clinical remission in human depression. In the present model, glia are therefore positioned as exploratory translator mechanisms between the acute postictal state and later consolidation, not as validated therapeutic mediators.

At present, the weakest evidentiary point is not the existence of individual postictal, endocrine, inflammatory, neurovascular, network, or plasticity changes, but the assumption that these changes form a coordinated, clinically predictive window. This assumption remains to be tested directly in prospective multimodal ECT cohorts.

Even if the postictal-window index proves biologically informative, its clinical translation will depend on whether a simplified version can be measured reliably, affordably, and reproducibly across ECT services.

## 11 Conclusion

ECT remains one of psychiatry’s strongest demonstrations that severe depression can be altered by a controlled biological intervention. Its mechanism has remained elusive partly because the field has repeatedly searched for a primary pathway: a transmitter, a hormone, a growth factor, a structure, a network, or a seizure metric. Severe depression is rarely maintained by one pathway. ECT is unlikely to work through one pathway.

We propose that the antidepressant action of ECT may depend on the repeated induction of a coordinated postictal systems window. The seizure triggers the treatment but is not identical with the clinically relevant event. The core candidate is the quality of postictal recovery: its amplitude, coordination, anatomical distribution, and duration. Endocrine, immune-metabolic, glial, trophic, structural, epigenetic, and BBB/NVU processes are not treated as equivalent mechanisms, but are positioned according to timing, evidentiary strength, and proposed role in the hierarchy.

The model does not replace neurotransmitter, endocrine, immune, vascular, network, or plasticity accounts of ECT. It orders them in time and rank. Its scientific value will depend on whether this ordering improves prediction, clarifies cognitive risk, and guides technique optimization. Its clinical value will depend on whether it helps psychiatrists understand ECT not as the crude production of a seizure, but as a controlled postictal systems intervention in severe depressive illness.

## Data Availability

The original contributions presented in the study are included in the article/supplementary material. Further inquiries can be directed to the corresponding author.
